# Infant urinary tract infection in Sweden — A national study of current diagnostic procedures, imaging and treatment

**DOI:** 10.1007/s00467-024-06415-4

**Published:** 2024-07-15

**Authors:** Magnus Lindén, Therese Rosenblad, Karin Rosenborg, Sverker Hansson, Per Brandström

**Affiliations:** 1 Department of Pediatrics, Halland Hospital, Halmstad, Sweden; 2 Department of Pediatrics, Institute of Clinical Sciences, Sahlgrenska Academy, University of Gothenburg, Gothenburg, Sweden; 3 Department of Pediatrics, Lund Children’s Hospital, Lund, Sweden; 4 Division of Microbiology, Immunology and Glycobiology, Institute of Laboratory Medicine, Lund University, Lund, Sweden; 5 Sachs’ Children and Youth Hospital, Södersjukhuset, Stockholm, Sweden; 6 Pediatric Uro-Nephrology Center, The Queen Silvia Children’s Hospital, Gothenburg, Sweden

**Keywords:** Urinary tract infection, Infant, Urine sampling, Vesicoureteral reflux, Clinical guidelines, Kidney damage

## Abstract

**Background:**

Urinary tract infection (UTI) in infants is a common, potentially life-threatening bacterial infection, and must be managed carefully through the entire chain of care from diagnosis, choice of treatment, follow-up and risk stratification of future complications. This Swedish nationwide study of infant UTI was conducted to evaluate the current management of infant UTI, yield of investigations and the Swedish UTI guidelines’ ability to detect abnormalities of importance in the urinary tract.

**Methods:**

Infants < 1 year with a first episode of UTI were included in a prospective multicenter study. Treatment and follow-up were provided by local pediatricians. Clinical and laboratory findings and imaging results were reported to the coordinating center. The current management and results were compared with a previous Swedish study.

**Results:**

One thousand three hundred six infants were included. Urine sampling was performed with clean catch technique in 93% of patients. Initial oral antibiotic treatment was used in 63%, predominantly third generation cephalosporines. Permanent kidney abnormalities were found in 10% and dilating vesicoureteral reflux (VUR) in 8%. Higher rates of male gender, non-*E. coli* infection and ultrasound dilatation were seen in infants < 1 month. UTI recurrences were reported in 18%.

**Conclusions:**

Infant UTI is still generating a considerable amount of follow-up examinations. There is a significant shift towards clean catch as the main urine sampling method. Voiding cystourethrography is performed less frequently reducing the findings of low grade VUR. The incidence of renal scarring is comparable with earlier studies which suggests that the Swedish guidelines are able to identify individuals with risk for long-term complications.

**Graphical abstract:**

A higher resolution version of the Graphical abstract is available as Supplementary information

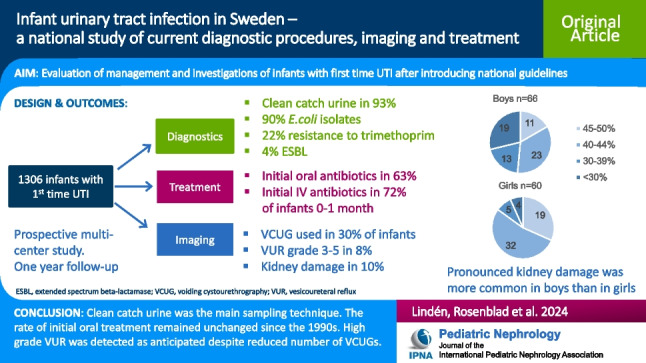

**Supplementary Information:**

The online version contains supplementary material available at 10.1007/s00467-024-06415-4.

## Introduction

Urinary tract infections (UTIs) are among the most common bacterial infections in childhood, affecting 2% of infants*,* creating a high number of patients to be treated throughout pediatric emergency units [1]. Diagnostic procedures are especially challenging during the first year of life, as symptoms and signs of disease are unspecific. Prompt treatment and investigations to identify children at risk of short and long-term complications are recommended in guidelines from different countries and various professional bodies around the world [2–7]. The burden of post infection examinations, however, has been considerable and the optimal algorithm to investigate young children with UTI and how to predict future risk of kidney-related morbidity are still matters of debate.

Children with vesicoureteral reflux (VUR) or other congenital anomalies of the kidneys and urinary tract are more prone to febrile UTIs. However, the majority of infants with febrile UTI do not have VUR or malformations and their UTI susceptibility is not fully understood. A subset of infants with a first UTI develop recurrent infections (14–21%), with increased risk of acquired kidney damage [8–11]. The rate of recurrent UTI is as high as over 50% in children with certain risk factors such as bladder and bowel dysfunction and dilating VUR [12, 13]. While historically much effort was put into detecting VUR after a first febrile UTI, focus has now shifted towards identifying susceptibility factors associated with severe acute infections, recurrent UTIs and renal scarring [14, 15].

The current national Swedish guidelines for management of children with febrile UTI launched in 2013 were aimed to reduce radiologic imaging in children under 2 years of age and, in particular, the number of voiding cystourethrographies (VCUGs) [16]. Reducing the investigations of infants with a first febrile UTI, however, may increase the risk of not identifying children at risk of kidney related morbidity or anomalies of the urinary tract.

The primary purpose of this paper was to give an updated overview of infant UTI management in Sweden, by describing the study population, the basis of diagnosis, bacterial findings and antimicrobial resistance, treatment, imaging findings and recurrence rate. A secondary purpose was to evaluate the current clinical guidelines and their ability to detect high grade VUR and permanent kidney damage.

## Patients and methods

We performed a prospective, observational, nationwide multicenter study of infant UTI in Sweden. All pediatric departments in the country were invited to participate and 29 out of 33 centers contributed to patient recruitment between March 20, 2017 and September 30, 2019. Infants < 1 year of age presenting with a first episode of suspected UTI were eligible for inclusion at the initiation of antibiotic treatment. Exclusion criteria were previous UTI, myelomeningocele, ongoing bladder catheterization and overt urogenital malformations. The diagnostic evaluation, treatment and further management were based on clinical judgement of the managing physician supported by the Swedish national guidelines. If the presumptive UTI diagnosis was abandoned after inclusion, the case was excluded. Infants with negative urine culture, missing culture or with mixed bacterial growth, yet treated as UTI, were also excluded but described below in a separate analysis.

A clinical report form was used for clinical data and patient characteristics from the acute episode including maximum recorded temperature, duration of fever (days) prior to starting treatment, circumcision status (boys), type of antibiotic treatment and administration mode, and results from investigations. A second report was registered 1 year after the index UTI including information on recurrent febrile UTIs, use and duration of antimicrobial prophylaxis and further imaging performed after the initial investigations.

According to the Swedish pediatric UTI guidelines, basic investigation included urine dipstick, urine culture, plasma creatinine, C-reactive protein (CRP) and renal and bladder ultrasonography (RBUS). Empiric treatment was recommended to commence intravenously with a third-generation cephalosporin and/or an aminoglycoside in severely ill children or those unable to take oral treatment. All other children were recommended an oral third generation cephalosporin, while trimethoprim/sulphamethoxazole was to be used only after the resistance pattern was known. An antibiotic course of 10 days was recommended in febrile UTI. Risk factors guiding further imaging of the urinary tract in infants were CRP ≥ 70 mg/L, creatinine > 30 µmol/L, non-*E. coli* infection and dilatation on RBUS with anteroposterior diameter (APD) ≥ 10 mm. For infants with one or more risk factors, 99mTc-dimercaptosuccinic acid (DMSA) scintigraphy was recommended to detect kidney involvement and/or congenital anomalies, and a follow-up DMSA scan for detection of permanent kidney injury in those with abnormal findings on the initial DMSA. 99mTc-mercaptoacetyltriglycine (MAG3) renography was used in some infants with dilatation of renal pelvis or ureter on RBUS. VCUG was limited to infants with dilatation on RBUS or uptake defects on DMSA scintigraphy.

### Laboratory tests

Blood and urine samples were analyzed at the local clinical laboratories and included plasma or serum CRP (maximum reported value during the acute phase of infection), plasma creatinine, urine dip stick results for nitrite and leukocyte esterase. A urine leukocyte esterase (U-Le) test was graded 0–4, where ≥ 1 + , corresponding to ≥ 15 cells/µL, was considered as pyuria.

Urine sampling methods were reported as suprapubic aspiration (SPA), catheter, clean catch or bag sample and urine cultures by bacterial species and number of colony forming units (CFUs) in intervals of < 10,000, 10,000 to < 100,000 and ≥ 100,000 CFU/mL. Antimicrobial resistance to trimethoprim, nitrofurantoin, mecillinam, ceftibuten, cefadroxil or other antibiotics was reported.

### Imaging studies

Imaging studies (RBUS, VCUG, DMSA and MAG3 scans) were performed at the local hospital and reported to the coordinating center. DMSA scans were considered abnormal when a focal uptake defect was detected or a relative function was below 45%. If both kidneys were affected, the kidney with the lowest relative function was used to characterize the patient. An abnormal DMSA scan 6 months or more after the index UTI was used to diagnose permanent kidney damage. Infants with a normal first DMSA scan and without recurrences were considered to have normal kidneys at follow-up [17]. When MAG3 renography was used, it was assessed with the same criteria.

VUR detected on VCUG was graded 1–5 according to recommendations by the International Reflux Study in Children [18]. In case of bilateral VUR, the highest grade was used to characterize the patient.

### Statistics

Statistical analyses were performed using SAS software version 9.4 (SAS Institute Inc., Cary, NC, USA). For normally distributed variables, mean and standard deviation were presented as descriptive data, for skewed variables median and interquartile range (IQR) were presented, and frequency and percent for categorical variables. Fisher’s exact test was used to compare two groups with dichotomous variables; the *χ*^2^ test was used for non-ordered categorical variables; Mantel–Haenszel *χ*^2^ trend test for ordered categorical variables, a two-sample *t*-test for normally distributed continuous variables and a Mann–Whitney *U*-test for skewed continuous variables. Univariable and multivariable logistic regression, based on forward selection, was applied when studying risk factors for recurrent UTI. Odds ratios (OR) with 95% CI and associated p-values are presented. The prediction ability was described by area under the receiver operating characteristic (ROC) curve, called c-statistics. All tests were 2-tailed and evaluated at the 0.05 significance level following a Bonferroni–Holm adjustment.

## Results

**Fig. 1:**
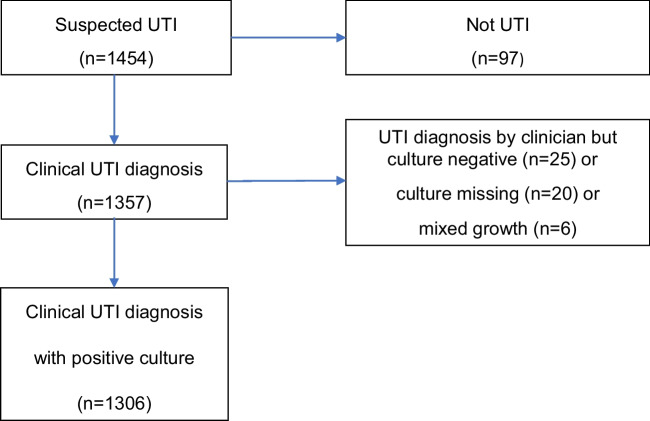
Flow diagram of the study population

The study enrolled 1454 infants (Fig. 1). Ninety-seven infants (7%) were diagnosed with other conditions and 1357 were judged and treated as having UTI by the managing pediatrician. Of the infants managed as UTI by the clinician, 25 were considered to have UTI despite negative urine cultures, in 20 cases urine culture was missing and in six there was mixed growth. The remaining 1306 (96%) cases were confirmed as UTI with positive urine cultures, constituting the population for further analysis. There were 601 boys (46%) with a median age of 2.7 months (IQR 1.3 to 4.7) and 705 girls (54%) with a median age of 5.6 months (IQR 2.9 to 8.4), *p* < 0.001. There was a male predominance in the first 4 months of life, thereafter UTI was more frequent in girls. Circumcision was reported in one boy.

### Character of UTI

**Fig. 2:**
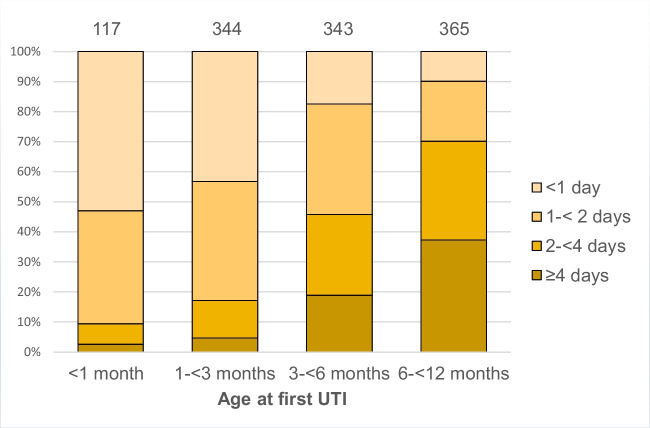
Fever duration prior to start of treatment, according to age in 1169 infants with febrile UTI (*p* < 0.001, significant following Bonferroni–Holm adjustment)

The demographics and clinical and laboratory data for boys and girls, respectively, are shown in Table 1. Maximum temperature during the acute phase of infection was recorded in 1270 patients and fever ≥ 38 °C was reported in 1190 infants (94%). Mean temperatures for boys and girls were 39.0 C° (± 0.9) and 39.3 C° (± 0.9), respectively (*p* < 0.001). The duration of fever before initiation of treatment increased with age (*p* < 0.001) and was less than 1 day in 26% of the febrile patients **(**Fig. 2).

**Table 1:** Demographics, clinical characteristics and laboratory findings at enrolment in 1306 infants with first time UTI

	Total	Boys *n* = 601 (46%)	Girls *n* = 705 (54%)	*p*-value
Age in months median (IQR)	4.0 (2.0–7.0) *n* = 1306	2.7 (1.3–4.7)	5.6 (2.9–8.4)	< 0.001*
Temperature (°C) mean (std)	39.2 (0.9) *n* = 1270	39.0 (0.9)	39.3 (0.9)	< 0.001*
Urine sampling method	*n*=1243			0.33
Clean catch	1150 (92.5%)	524 (92.1%)	626 (92.9%)	
SPA	62 (5.0%)	34 (6.0%)	28 (4.2%)	
Cath	20 (1.6%)	7 (1.2%)	13 (1.9%)	
Bag	11 (0.9%)	4 (0.7%)	7 (1.0%)	
U-nitrite positive	499 (38.5%), *n* = 1295	227 (38.2%)	272 (38.9%)	0.819
Pyuria (U-Le positive ≥ 1 +)	1263 (97.4%)	578 (96.5%)	685 (98.1%)	0.081
U-Le-test 0	34 (2.6%)	21 (3.5%)	13 (1.9%)	
+	67 (5.2%)	31 (5.2%)	36 (5.2%)	
2 +	271 (20.9%)	109 (18.2%)	162 (23.2%)	
3 +	441 (34.0%)	197 (32.9%)	244 (35.0%)	
4 +	484 (37.3%) *n* = 1297	241 (40.2%)	243 (34.8%)	
CRP (mg/L) median (IQR)	62 (28–109) *n* = 1294	58 (26–99)	67 (29–118)	0.003
Creatinine (µmol/L) median (IQR)	21 (18–24) *n* = 1256	21 (18–25)	20 (17–24)	< 0.001*
Creatinine > 30 µmol/L	79 (6.3%) *n* = 1256	46 (7.9%)	33 (4.9%)	0.027

*IQR* Interquartile range, *std* standard deviation, *SPA* suprapubic aspiration, *Cath* catheter, *U-Le* urine leukocyte esterase, *CRP* C-reactive protein

^*^Significant following Bonferroni-Holm adjustment

### Sampling method and urinalysis

Data on the urine sampling method was obtained in 1243 cases. A clean catch specimen was used in 1150 (92.5%), SPA in 62 (5.0%), catheter in 20 (1.6%) and collecting bag in 11 (0.9%). SPA was rarely performed except in the two largest centers. There were no significant differences between sampling methods regarding urine nitrite test, pyuria, bacterial numbers or proportion of non-*E. coli.*

A U-Le test was reported positive in 1263/1297 cases (97.4%) while nitrite was positive in 499/1295 cases (38.5%), both with no significant gender difference (*p* = 0.081 and *p* = 0.819, respectively).

### Bacterial typing and antimicrobial sensitivity

**Fig. 3:**
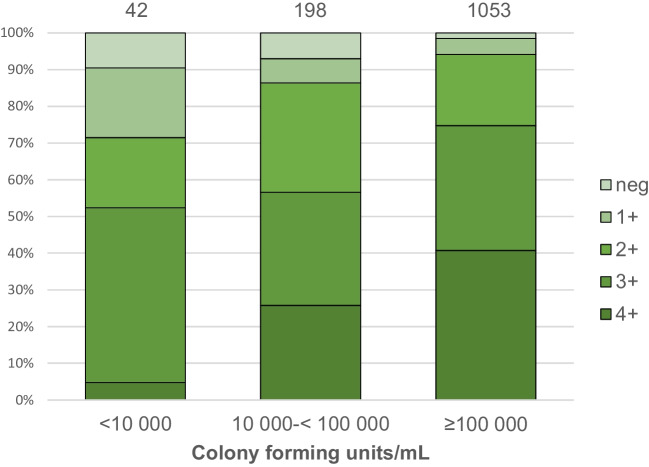
Grade of pyuria on urine leukocyte esterase test according to bacterial number in urine culture in 1293 infants. 1 + corresponds to ≥ 15 cells/µL (*p* < 0.001, significant following Bonferroni–Holm adjustment)

Data on bacterial counts, available in 1301 urine cultures, showed 1058 (81%) with ≥ 100,000 CFU/mL, 201 (15%) with 10,000 to < 100,000 CFU/mL and 42 (3%) with < 10,000 CFU/mL. The predominating bacterial species was *E. coli,* reported in 1169 infants (90%). Data on bacteria and the corresponding findings in laboratory parameters and RBUS are summarized in Table 2. There were significant differences in bacterial counts, CRP level, rate of positive nitrite, pyuria, creatinine > 30 µmol/L and rate of dilatation on RBUS between infants with *E. coli* and non-*E. coli* infections. The higher grade of pyuria correlated with the higher bacterial count (Fig. 3).

**Table 2:** Bacterial species in urine cultures, gender, age, laboratory findings and collecting system dilatation on RBUS in 1306 infants with *E. coli* and non-*E. coli* UTI

	*E. coli* n = 1169 (90%)	Non-*E. coli* n = 137 (10%)	*p*-value
Non-*E. coli* isolates		Klebsiella 69 (5.3%) Enterococcus faecalis 18 (1.4%) Enterobacter 15 (1.1%) Citrobacter 13 (1.0%) Group B streptococcus 10 (0.8%) Other^a^ 12 (0.9%)	
Gender boy/girl	529 (45.3%)/640 (54.7%)	72 (52.6%)/65 (47.4%)	0.123
Age at UTI, months median (IQR)	4.1 (2.1–7.1)	3.1 (1.8–5.6)	0.002
Nitrite positive	469/1158 (40.5%)	30/137 (21.9%)	< 0.001*
U-Le positive	1140/1161 (98.2%)	123/136 (90.4%)	< 0.001*
Bacterial count ≥ 100,000 CFU/mL	960/1165 (82.4%)	98/136 (72.1%)	0.005
CRP median (mg/L) (IQR)	64 (*n* = 1158) (31–110)	46 (*n* = 136) (10–99)	0.001*
Creatinine median (µmol/L) (IQR)	20 (*n* = 1125) (18–24)	22 (*n* = 131) (19–27)	< 0.001*
Creatinine > 30 µmol/L	59/1125 (5.2%)	20/131 (15.3%)	< 0.001*
Dilatation on RBUS	138/1163 (11.9%)	42/137 (30.7%)	< 0.001*

*IQR*, Interquartile range; *U-Le*, urine leukocyte esterase; *CRP*, C-reactive protein; *RBUS*, renal and bladder ultrasound

^a^*Proteus mirabilis* 4, *Aerococcus urinae* 2, *Staphylococcus aureus* 2, Coagulase-negative staphylococcus 2, *Haemophilus influenzae* 1, *Pseudomonas aeruginosa* 1

^*^Significant following Bonferroni–Holm adjustment

Trimethoprim resistance was detected in 22% of the *E. coli* isolates, followed by amoxicillin/clavulanic acid 8%, cefadroxil 4%, ceftibuten 2%, mecillinam 2%, and nitrofurantoin 0.2%. Extended spectrum beta-lactamase production (ESBL) was found in 4%.

### Blood tests

The median CRP was 62 mg/L (IQR 28 to 109) and was higher in girls than in boys, 67 mg/L (IQR 29 to 118) and 58 mg/L (IQR 26 to 99), respectively (*p* = 0.003). Creatinine > 30 µmol/L was seen more often in boys, 46/579 (7.9%), than in girls, 33/677 (4.9%), *p* = 0.027, see Table 1.

### Treatment

Treatment was initiated with intravenous antibiotics in 484 of 1305 patients (37%, information missing in one patient) and oral antibiotics in 821 (63%) of which 22 (3%) were temporarily switched to intravenous treatment. The use of initial oral antibiotics varied between centers and was higher in the two largest centers, 84% and 82% respectively, compared to 54% among the remaining centers. The younger infants were treated with intravenous antibiotics more frequently than the older ones: < 1 month 72%, 1– < 3 months 53%, 3– < 6 months 28% and 6– < 12 months 23% (*p* < 0.001). Initial intravenous treatment was changed to oral within three days in 82% of the cases. A change from the initial antibiotic therapy due to antimicrobial resistance was reported in 30 patients (2%).

The three most commonly used oral antibiotics as first line treatment were third generation cephalosporines in 718 (87%), trimethoprim/sulfamethoxazole in 77 (9%) and amoxicillin/clavulanic acid in 9 (1%). Among the initially intravenously treated infants, cefotaxime was used in 469 patients (97%) either as single therapy or combined with another agent (22 patients).

### Imaging of the urinary tract

#### Ultrasound

RBUS was performed in 1300/1306 infants. The APD of the pelvis was not consistently reported. However, dilatation of the urinary tract was reported in 180 infants (14%), more frequently in boys, 115/598 (19%), than in girls, 65/702 (9%), *p* < 0.001. Bilateral dilatation was reported in 35 infants (3%). Kidney duplication was seen in 22 cases, multicystic dysplasia in three, unilateral aplastic kidney and horseshoe kidney in two each, and crossed kidney ectopia and kidney tumor in one each.

#### VCUG

VCUG was performed in 390 infants (30%) and VUR was detected in 147 (38%) of these; grade 1 in 10 (3%), grade 2 in 27 (7%), grade 3 in 32 (8%), grade 4 in 55 (14%) and grade 5 in 23 infants (6%). VUR grade 3–5 was more frequent in infants with dilatation on RBUS than without dilatation, 49/136 (36%) vs. 61/254 (24%), *p* = 0.013, not significant following Bonferroni–Holm adjustment. However, 61/110 (56%) of infants with VUR grade 3–5 had no dilatation on RBUS. Of the 23 infants with VUR grade 5, seven (30%) had no dilatation on RBUS. Bilateral dilating VUR was found in 40 infants and posterior urethral valves in seven boys.

#### Scintigraphy

**Fig. 4:**
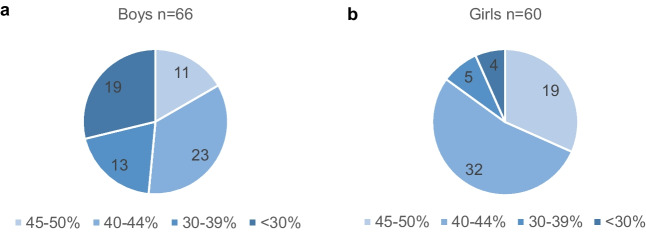
Relative function of most affected kidney in (**a**) 66 boys compared to (**b**) 60 girls with permanent kidney damage on DMSA or MAG3 scintigraphy. In five cases of bilateral damage, the most severely affected kidney is presented (*p* < 0.001, significant following Bonferroni–Holm adjustment)

Scintigraphy was performed in 836 infants (774 DMSA and 62 MAG3 scans) of which five were performed prior to the first UTI due to antenatally diagnosed hydronephrosis and 363 within 30 days after the acute infection. Among these 363 scans, 148 (41%) were abnormal. In 213 infants, at least one more scintigraphy was performed during follow-up. Scintigraphy data for evaluation of permanent kidney defects after 6 months was available in 648 infants, of which 126 (19%) were abnormal (33 of the follow-up scans were MAG3). Among these infants, five had bilateral damage. The degree of kidney impairment categorized by the relative function of the most affected kidney was more severe in boys than in girls (*p* < 0.001), Fig. 4. There was no difference in the rate of permanent kidney damage between infants with intravenous antibiotic treatment, 70/316 (22%), and oral treatment only, 56/332 (17%), *p* = 0.092.

### Follow-up after first UTI

At least one recurrent febrile UTI episode was reported in 229/1290 infants (18%), 86 (14%) boys and 143 (21%) girls. Significant risk factors for recurrent UTI following Bonferroni-Holm adjustment were non-*E. coli* bacteria at first UTI and dilatation on RBUS. In the multivariable model, female sex and dilatation on RBUS were independent risk factors, see Table 3.

**Table 3:** Univariable and multivariable logistic regression of risk factors for febrile recurrent UTI ^a^. Total recurrence rate 18%

				Univariable models			Multivariable model	
Variable	Values	Missing data	*n* (%) events	OR (95% CI)	*p*-value	AUC	OR (95% CI)	*p*-value
Age in months (by 1 month increase)	≤ median	0	123 (18.9%)					
	> median	0	106 (16.6%)	0.97 (0.93–1.02)	0.23	0.52	0.94 (0.89–0.99)	0.014
Sex	Boys	0	86 (14.4%)	Ref			Ref	
	Girls	0	143 (20.6%)	1.54 (1.15–2.07)	0.0037	0.55	2.07 (1.49–2.88)	< .0001*
Bacteria at first UTI	*E. coli*	0	189 (16.4%)	Ref			Ref	
	Non-*E. coli*	0	40 (29.4%)	2.13 (1.43–3.18)	0.0002*	0.54	1.83 (1.20–2.79)	0.0053
Dilatation on RBUS (ref No)	No	6	170 (15.4%)	Ref			Ref	
	Yes	6	59 (33.0%)	2.70 (1.90–3.84)	< .0001*	0.57	2.77 (1.91–4.02)	< .0001*
CRP (mg/L) (by 1 mg/dL increase)	≤ median	12	98 (15.2%)					
	> median	12	130 (20.5%)	1.00 (1.00–1.00)	0.10	0.56		
CRP (mg/L)	CRP < 70	12	108 (15.2%)	Ref			Ref	
	CRP ≥ 70	12	120 (21.1%)	1.49 (1.12–1.99)	0.0062	0.55	1.51 (1.12–2.04)	0.0065

*rUTI*, recurrent urinary tract infection; *RBUS*, renal and bladder ultrasound; *CRP*, C-reactive protein; *OR*, odds ratio; *CI*, confidence interval; *AUC*, area under the curve

^a^Data on recurrent UTI missing in 16 infants

^*^Significant following Bonferroni–Holm adjustment. Area under receiver operating characteristic curve (AUC) for the multivariable model was 0.66

Antimicrobial prophylaxis was prescribed to 237 of 1293 infants (18%), 29 (2%) for < 2 months and 206 (16%, 112 boys and 94 girls) for ≥ 2 months (no information on duration in two). Surgical interventions by a pediatric urologist were performed in 60 patients, for details see the Supplementary Table S1. One death was reported during the study period in conjunction with a recurrent UTI.

### Influence of age

There were 140 children below 1 month of age included in the study. They differed in many aspects compared to older infants, with a pronounced male predominance, lower temperature, higher rate of non-*E. coli*, higher creatinine and a higher rate of dilation on RBUS, Table 4. The gender difference in the total study population with a higher rate of raised creatinine in boys was not seen when infants < 1 month were excluded.

**Table 4:** Clinical, laboratory and imaging findings related to age

	< 1 month^a^ *n* = 140	≥ 1 month *n* = 1166	*p*-value
Male (%)	112 (80.0%)	489 (41.9%)	< 0.001
Temperature ≥ 38°C	120/138 (87.0%)	1070/1132 (95.5%)	0.002
Urine sampling method			
Clean catch	106 (82.8%)	1044 (93.6%)	
SPA	15 (11.7%)	47 (4.2%)	
Catheter	3 (2.3%)	17 (1.5%)	
Bag	4 (3.1%) *n* = 128	7 (0.6%) *n* = 1115	
Nitrite positive	58/139 (41.7%)	441/1156 (38.1%)	0.408
U-Le positive (≥ 1 +)	130 (92.9%)	1133/1157 (97.9%)	0.002
Non-*E. coli*	22 (15.7%)	115 (9.9%)	0.040
Bacterial count ≥ 100,000 CFU/mL	119/138 (86.2%)	939/1163 (80.7%)	0.133
CRP (mg/L) Median (IQR)	59.5 (24–102)	63 (28–110) *n* = 1154	0.236
Creatinine > 30 µmol/L	34/137 (24.8%)	45/1119 (4.0%)	< 0.001
Intravenous antibiotics	101 (72.1%)	405/1165 (34.8%)	< 0.001
Dilatation on RBUS	30 (21.4%)	150/1160 (12.9%)	< 0.001
VUR grade 3–5	17/57 (29.8%)	93/333 (27.9%)	0.752
Abnormal kidney scan six months after initial UTI	18/84 (21.4%)	108/564 (19.1%)	0.658

*SPA* suprapubic aspiration, *U-Le* urine leukocyte esterase, *CRP* C-reactive protein, *IQR* interquartile range, *RBUS* renal and bladder ultrasound, *VUR* vesicoureteral reflux

^a^Age in days: median 19, minimum 5, IQR 11

### Separate analysis of infants without confirmative urine culture

In 25 infants treated and managed as having UTI, the urine culture yielded no bacterial growth. These children were severely ill and in the majority antibiotic treatment was given before urine sampling. A comparison between these infants and those with bacterial growth of a single pathogen is shown in Table 5. The group with negative urine culture were younger and had a higher rate of creatinine > 30 µmol/L and dilatation on RBUS than the culture positive group. Among these 25 infants, two were subject to heminephrectomy due to obstruction and two had endoscopic treatment for dilating VUR and recurrent UTIs. For the 26 patients excluded due to urine culture missing or showing mixed growth, but treated as UTI, there were no such differences compared to the study population.

**Table 5:** Clinical and laboratory parameters and imaging studies in infants with culture verified vs. culture negative UTI and vs. infants with missing culture (*n* = 20) or mixed growth (*n* = 6), yet treated as UTI

	Culture pos *n* = 1306	Culture neg *n* = 25	Culture missing or mixed growth *n* = 26	*p*-value^a^	*p*-value^b^
Male	601 (46%)	17 (68%)	11 (42%)	0.041	0.843
Age median (months) (IQR)	4.0 (2.0–7.0)	1.5 (0.9–2.8)	3.8 (2.9–5.9)	< 0.001*	0.830
Temperature (°C) mean (std)	39.2 (0.9) *n* = 1270	38.7 (0.7) *n* = 24	39.1 (0.9) *n* = 24	0.014	0.798
U-nitrite pos	499/1295 (39%)	12/24 (50%)	10 (39%)	0.292	1.000
CRP median (mg/L) (IQR)	62 (28–109) *n* = 1294	79 (52–107)	66 (50–112)	0.081	0.413
Creatinine > 30 µmol/L	79/1256 (6.3%)	7/23 (30%)	3 (12%)	< 0.001*	0.229
Dilatation on RBUS	180/1300 (14%)	10 (40%)	5 (19%)	0.001*	0.394
VUR grade 3–5	110/390 (28%)	7/14 (50%)	2/8 (25%)	0.128	1.000
Abnormal kidney scan 6 months after initial UTI	126/648 (19%)	4/11 (36%)	1/12 (8%)	0.241	0.478

*IQR*, interquartile range; *std*, standard deviation; *CRP*, C-reactive protein; *IV*, intravenous; *RBUS*, renal and bladder ultrasound; *VUR*, vesicoureteral reflux

^a^*p*-value in culture neg vs. culture pos

^b^*p*-value in culture missing or mixed growth vs. culture pos

^*^Significant following Bonferroni–Holm adjustment

## Discussion

This nationwide study of 1306 infants with UTI provides a comprehensive overview of current diagnostic procedures, treatment, and management in Swedish pediatric care. According to national recommendations, infants with a suspected UTI are referred to and managed by pediatricians and we believe that the study population is representative for this age group.

Compared to the Swedish quality assurance project in the mid-1990s, a nationwide study of children < 2 years with first time UTI, our study revealed a remarkable shift in urine sampling method in favor of the clean catch technique, practiced in 93% of cases compared to only 11% in the 1990s, while bag samples, previously used in 50% of urine cultures, have virtually disappeared [19]. Bag samples, known to carry a high risk of contamination, are rarely used nowadays. However, it is surprising that SPA was infrequently performed in this population of young infants despite being recommended in the national guidelines. Clearly, clean catch urine has become the method of choice in pediatric specialist care in Sweden, a trend seen also in other parts of the world. This sampling technique has been proposed as an alternative to invasive techniques for infants in good clinical condition by the Italian, Indian, Australian and Swiss guidelines [5, 7, 20, 21] and is the recommended method in the NICE guidelines [2]. Clean catch urine combined with a standardized stimulation technique has shown similar contamination risk as urethral catheterization [22] while others advocate SPA or catheter specimens to avoid contamination [23].

Similar to other reports, we found non-*E. coli* in 10% of the infants, with comparable rates of *Enterococcus* and *Klebsiella* species, known to be associated with lower bacterial counts and less pyuria [24–26]. Eighteen percent of infants in our cohort had bacterial numbers less than 100,000 CFU/mL. This was in line with the frequency of 19–20% found in other studies [27–29]. These results indicate that disregarding urine cultures with low bacterial counts or low-grade pyuria in this age group could lead to a missed or delayed diagnosis and treatment.

From an international perspective, our study showed a low frequency of resistant uropathogens. A meta-analysis of antimicrobial resistance in children 0–5 years treated for UTI in primary care showed that the pooled prevalence of resistance in *E. coli* isolates was 29.8% for trimethoprim sulphamethoxazole, 9.6% for amoxicillin/clavulanic acid, and 4.9% for third generation cephalosporins [30].

Initial intravenous treatment was given to 37% of infants and was more frequent in younger age groups. Although 40% of the study subjects were younger than 3 months, we anticipated a more frequent use of oral antibiotics as initial treatment. The use of oral antibiotics for UTI in Swedish pediatric care has not changed since the 1990s despite evidence of oral antibiotic treatment being equally effective as intravenous administration in well appearing children [31, 32]*.* In the former Swedish study, the proportions of initial intravenous treatment in age groups 0– < 3 months, 3– < 6 months and 6– < 12 months were 51%, 31% and 24%, respectively, and in the current study 56%, 28%, and 21% [19]. In our study, there were similar rates of permanent kidney damage between oral and intravenous treatment groups, thereby supporting the evidence of safe oral antibiotic treatment in infant UTI. The practice of initial empirical treatment with oral antibiotics in infants in our study, however, was in sharp contrast to the standard practice of care in Japan. This was presented in a recent study on children aged < 36 months with UTI where almost all patients were hospitalized and initially treated with intravenous antibiotics and 81% of the patients were switched to oral antibiotics as late as day 4 to 8 of hospitalization [33].

Long-term antimicrobial prophylaxis was used in 16% of the patients, similar to the 1990s Swedish quality assurance study where it was used in 20% of the cases [19]. However, the use of prophylaxis while awaiting the initial imaging to be performed, has dropped from 79 to only 2%.

During the study period, prenatal ultrasound screening was offered to pregnant women at gestational week 12–14 (combined ultrasound and blood test for chromosomal anomalies) and week 18–19 (including organ screening) in most parts of the country. We did not ask for the information on any prenatal ultrasound in this study. Recommendations in the national UTI guidelines were to perform RBUS in all infants with first time UTI, justified by the need to identify children with major congenital abnormalities or obstruction of the kidneys and urinary tract. We used high creatinine as a risk factor that could indicate a more severely ill child with prerenal acute kidney injury, congenital hypo/dysplasia, known to be more common in boys, or kidneys that are more vulnerable to consequences of the infection. This should alert the caregiver to pay extra attention to that child and motivate further assessment and imaging.

Our study included a multivariable analysis of risk factors for recurrent UTI showing female gender as a significant independent risk factor. The higher recurrence rate in girls than in boys (21% vs. 14%) was in accordance with the Swedish reflux trial where recurrent UTIs were seen more frequently in girls (33%) than in boys (9%), although these children were all above 1 year of age [13]. Other studies on gender as a risk factor for recurrent UTI, however, are scarce and results not conclusive, partly due to small studies and diverging populations. A large pediatric primary care study by Conway et al. found no significant gender difference although children up to 6 years of age were included [34]. Neither could two smaller studies including younger children with first time UTI find significant differences in recurrence rate between males and females [35, 36].

The result of the Swedish quality assurance project [19] was a reflection of the management of infant UTI at that time, where most local guidelines were based on a bottom-up approach and kidney scarring was identified at follow-up with intravenous urography or DMSA scintigraphy. In comparison, we found a substantial reduction in the frequency of VCUGs in our study, 30% versus 85% in the 1990s. Furthermore, the use of intravenous urography has dropped from 25% of the patients to no urographies at all. On the other hand, 59% of children in our study were exposed to a DMSA scan, compared to 25% in the previous study and 13% had two DMSA scans during the study period. The radiation dose of a DMSA scan is approximately 0.7–1 mSv, equivalent to about 4–6 months of natural background radiation [37]. In light of the potential risks with exposure to ionizing radiation in early infancy it is prudent to continue the efforts of reducing the radiation burden for infants with UTI [38].

VUR grade 4 or 5, a major risk factor for scarring was detected in 78/1306 infants (6%) in our study population. In a meta-analysis by Shaik et al., where 1247 of 1280 children with a first UTI were examined by VCUG, 4% showed VUR grade 4 or 5 [39]. In a single center study of infants with UTI where VCUG was performed in all participants the detection rate of VUR grade 4 or 5 was 5% [40]*.* Our relatively high rate of VUR grade 4 and 5, despite only 30% of the cohort being subject to a VCUG indicates that the Swedish guidelines effectively identified infants with a high risk of dilating VUR.

Results from the DMSA and MAG3 scintigraphies identified permanent abnormalities of the kidneys in 126 of the 1306 infants (10%) which could result in further investigations or long-term follow-up. Since our study only had end point data for permanent kidney damage from 648 of the 1306 patients (50%) in the cohort, 10% is a minimum rate. Boys had significantly more severe kidney defects than girls. This yield is comparable to Hoberman et al., reporting 26 cases of renal damage after UTI in 275 children (9.5%) [17]. However, according to other studies, the expected rate of kidney scarring after a first UTI varies from 15 to up to 25% when discrete uptake defects on DMSA were also included [41, 42]. Furthermore, in a recent study of prophylaxis or no prophylaxis to infants with dilating VUR before any UTI, new parenchymal kidney defects occurred regardless of whether the children had a UTI or not during follow-up [43].

There were 25 infants excluded from the study, with no growth in the urine culture, yet diagnosed and treated as having UTI. Compared to the study population, these infants were younger and more likely to have affected kidney function by elevated creatinine (Table 4). Furthermore, dilatation on RBUS was more prevalent, as was dilating VUR and kidney damage although not reaching significance levels. These infants were reported to be ill appearing and, in the majority, a first dose of antibiotics was given prior to urine sampling. Thus, these infants comprise a clinically challenging group, although not fitting into the study requisite.

A limitation with the study was that imaging investigations and chemistry lab tests were analyzed and interpreted in the local laboratories with the risk of regional and individual differences or errors. There was no requirement of standardized protocol for the ultrasound examinations performed at the 29 different centers and reports often lacked data on APD. Yet, the rate of dilatation of the urinary tract in our study, 14%, was very similar to the 15% rate of dilatation found in a single center study with a standardized protocol on 290 infants with UTI [40].

The predominant use of clean catch urine sampling carries a risk of patients being included who did not suffer from a true UTI. In addition, as the guidelines did not define any specific limit for bacterial count, a few children with contaminated urine culture may have been falsely classified as having UTI. On the other hand, this makes the study more relevant for evaluating management and patient characteristics in every day clinical care of infants treated for UTI and evaluating the diagnostic yield, burden of investigations and functionality of clinical guidelines in routine pediatric practice.

From public demographic data and expected UTI incidence in infants, we estimate that there were roughly 5000 infants eligible for inclusion during the study period. However, there was no indication of systematic bias in the sample selection. A few centers in the country did not include any patients at all and when analyzing two of the largest centers, there was no difference in gender, age or temperature between the children included in the study and those not included.

In conclusion, this report describes current management of infants with first time UTI in pediatric centers in Sweden and provides data on the yield of the implemented investigations in a large and representative population. Since the 1990s, a major shift in urine collection method in favor of the clean catch technique was noted. Furthermore, the use of prophylactic antibiotics while awaiting investigation with VCUG, has virtually disappeared. Initial intravenous antibiotic treatment was surprisingly unchanged providing incentive for improvement by increased outpatient management or oral antibiotic use in inpatient care. This would render savings for the healthcare provider and less traumatic care.

The study results indicate that the Swedish UTI guidelines are able to identify important abnormalities such as permanent kidney damage and dilating VUR. We have reduced the number of VCUG considerably, however, the number of DMSA investigations on infants with UTI is still substantial. Our work continues with reducing the burden of investigations further, in particular radiation exposure, and developing individualized methods of risk assessment. The management of the very youngest infants should be more clearly addressed in future revisions of the guidelines.

## Supplementary Information

Below is the link to the electronic supplementary material.

Graphical abstract (PPTX 80 KB)

Supplementary file2 (DOCX 18 KB)

## Data Availability

The data set generated during and/or analyzed during the current study are available from the corresponding author on reasonable request.
