# Supplementary material for: Infant urinary tract infection in Sweden — A national study of current diagnostic procedures, imaging and treatment
Source: Pediatr Nephrol. 2024 Jul 15;39(11):3251–62. doi: 10.1007/s00467-024-06415-4 (PMC11413111; doi:10.1007/s00467-024-06415-4)
Supplement: Supplementary file 1 — Graphical abstract (PPTX 80 KB) [file 467_2024_6415_MOESM1_ESM.pptx]

## Slide 1
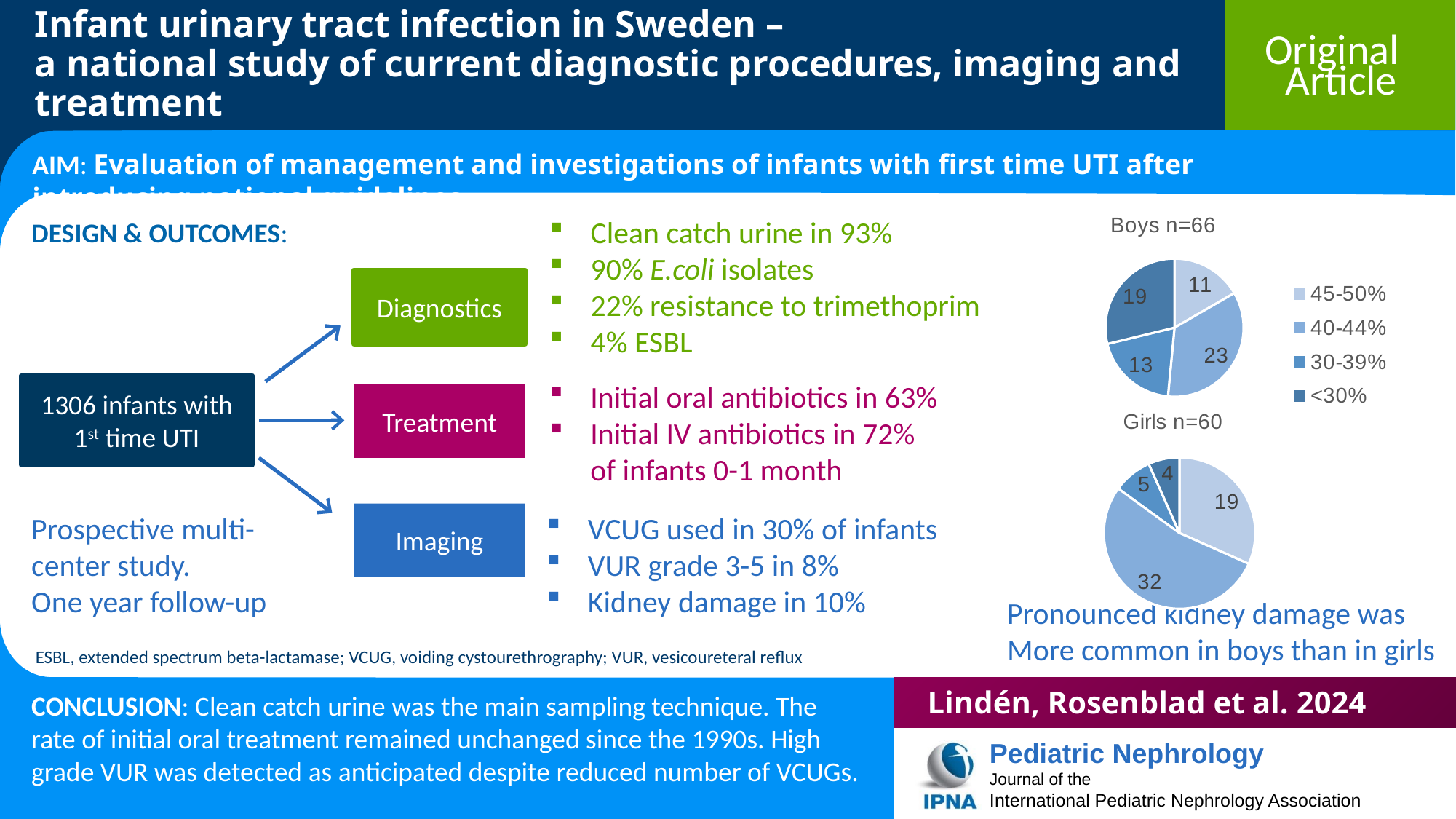

Infant urinary tract infection in Sweden –
a national study of current diagnostic procedures, imaging and treatment
AIM: Evaluation of management and investigations of infants with first time UTI after introducing national guidelines
### Chart:
| Category | Boys n=66 |
|---|---|
| 45-50% | 11.0 |
| 40-44% | 23.0 |
| 30-39% | 13.0 |
| <30% | 19.0 |Clean catch urine in 93%
90% E.coli isolates
22% resistance to trimethoprim
4% ESBL
DESIGN & OUTCOMES:
Diagnostics
Initial oral antibiotics in 63%
Initial IV antibiotics in 72% of infants 0-1 month
1306 infants with 1st time UTI
### Chart:
| Category | Girls n=60 |
|---|---|
| 45-50% | 19.0 |
| 40-44% | 32.0 |
| 30-39% | 5.0 |
| <30% | 4.0 |Treatment
Imaging
Prospective multi-
center study.
One year follow-up
VCUG used in 30% of infants
VUR grade 3-5 in 8%
Kidney damage in 10%
Pronounced kidney damage was
More common in boys than in girls
ESBL, extended spectrum beta-lactamase; VCUG, voiding cystourethrography; VUR, vesicoureteral reflux
Lindén, Rosenblad et al. 2024
CONCLUSION: Clean catch urine was the main sampling technique. The rate of initial oral treatment remained unchanged since the 1990s. High grade VUR was detected as anticipated despite reduced number of VCUGs.
