# Supplementary material for: Infant urinary tract infection in Sweden — A national study of current diagnostic procedures, imaging and treatment
Source: Pediatr Nephrol. 2024 Jul 15;39(11):3251–62. doi: 10.1007/s00467-024-06415-4 (PMC11413111; doi:10.1007/s00467-024-06415-4)
Supplement: Supplementary file 2 — Supplementary file2 (DOCX 18 KB) [file 467_2024_6415_MOESM2_ESM.docx]

|  | Number of performed procedures |
| --- | --- |
| Endoscopic treatment of VUR | 24^a^ |
| Neoimplantation of ureter | 4 |
| Nephrectomy | 3 |
| Heminephrectomy | 3 |
| Pyeloplasty | 6 |
| Posterior urethral valve ablation | 7 |
| Incision or resection of ureterocele | 5 |
| Circumcision due to recurrent UTI | 5^a^ |
| Urethral meatoplasty | 1 |
| Ureteral stent and balloon dilatation | 2 |
| Nephrolitiasis surgery | 1 |
| Total | **61^a^** |

Supplementary table

**Table S1** Procedures and surgery performed by pediatric urologists in 60 of 1306 infants with first time UTI

^a^One patient subject to both endoscopic treatment and circumcision
